# Tracheal necrosis following two‐stage thyroidectomy: A case report

**DOI:** 10.1002/ccr3.6245

**Published:** 2022-09-02

**Authors:** Taylor Colvin, Chris Selinsky

**Affiliations:** ^1^ Department of Otolaryngology OhioHealth‐Doctors Hospital Columbus Ohio USA; ^2^ Otolaryngology Department OhioHealth‐Doctors Hospital Columbus Ohio USA

**Keywords:** ear, nose and throat, general surgery, oncology, thyroidectomy, tracheal necrosis

## Abstract

Tracheal necrosis is a rare complication of a thyroidectomy, almost exclusively following the resection of invasive thyroid carcinoma with tracheal involvement. We report a case of delayed tracheal necrosis following a routine thyroidectomy for noninvasive papillary thyroid carcinoma likely secondary to a postoperative seroma. This complication was successfully managed with a temporary tracheostomy.

## Graphical abstract

A surgeon must be prepared to manage all possible complications from surgeries they offer to patients. Tracheal necrosis is a very uncommon complication of a thyroidectomy; however, this case demonstrates this as a possibility as well as a potential treatment plan in the event it does occur.

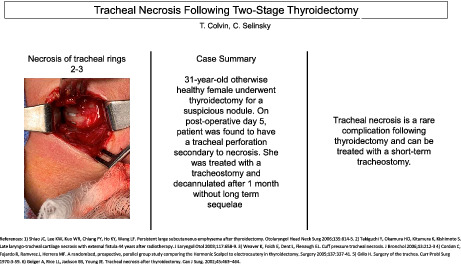

## 1 INDRODUCTION

When preparing a patient for a thyroidectomy, the more common complications are discussed with them prior to surgery, which may include recurrent nerve injury, temporary or permanent damage to parathyroid glands, and the need for additional surgeries. Following surgery for noninvasive thyroid carcinoma, tracheal necrosis is extremely rare with very few reports in the literature.

We report successful management of tracheal necrosis in a patient who underwent a thyroid lobectomy for potential papillary carcinoma, followed by a completion thyroidectomy as tissue examination revealed a 3.1 cm follicular variant papillary carcinoma.

## 2 CASE PRESENTATION

A 31‐year‐old woman with past medical history of anxiety presented with a thyroid nodule that was palpated during an annual checkup with her PCP. At the time of presentation, she was asymptomatic and denying symptoms of dysphagia, hoarseness of voice, and odynophagia. She does report a family history of thyroid carcinoma in her mother at 60 years old that was treated with thyroidectomy and radioactive iodine. Ultrasound and FNA were performed revealing a TR3 follicular neoplasm measuring 3.7 × 1.9 × 3.5 cm and pathology suspicious for follicular neoplasm. Pathology was then sent for molecular testing utilizing Afirma, a genomic sequencing classifier by Veracyte, which resulted as “suspicious for malignancy” indicating a 50% risk of malignancy. The patient was presented with options of a total thyroidectomy or a hemithyroidectomy, with patient electing the latter.

The patient was brought to the operating room for right hemithyroidectomy. She was intubated with a 7 mm inner diameter (10 mm outer diameter) electrode‐embedded EMG endotracheal tube, with the cuff inflated until no air leak detected, and precise cuff pressure was not documented. Dissection and hemostasis were achieved with bipolar cautery set at 20 watts for coagulation and a harmonic scalpel (ultrasonic shears by Ethicon) for hemostasis. Prior to closure, NIM‐Response 3.0 System by Medtronic was used to verify recurrent laryngeal nerve had not been violated during the procedure. There was no sign of adjacent tissue invasion of the tumor, minimal amount of bleeding was encountered during the procedure, and the only anatomical variant noted was the absence of a thyroid isthmus. Total intubation time for the procedure was 1 hour and 52 minutes. Final pathology revealed follicular variant papillary carcinoma with tumor measuring 3.1 cm with tumor capsule invasion, which was subsequently staged as pT2, pNx (stage 1). Seven days after the right hemithyroidectomy, due to pathology results patient underwent a completion thyroidectomy with the removal of any residual thyroid tissue on the right side. Procedure again was uneventful with minimal bleeding (approximate EBL 20 cc), followed by the removal of any residual thyroid tissue on the right side. Dissection method was equivalent to the first procedure with recurrent nerve integrity again confirmed with NIM‐Response monitoring system; however, total intubation time for the second procedure was 2 h and 59 min.

Immediate postoperative course was uncomplicated apart from a low PTH treated with temporary oral calcium and vitamin D supplementation. She was seen postoperatively in the clinic 3 days following completion thyroidectomy when mild drainage from the incision site was noted along with peri‐incisional erythema. Needle aspiration was performed removing 14 cc of serosanguineous fluid with patient expressing relief of pressure. She was prophylactically placed on cephalexin 500 mg 3 times per day. Five days later (8 days since completion thyroidectomy), she presented to our clinic with an open wound with air escape noted. Fiberoptic laryngoscopy was not performed in the clinic; however, the patient did not have complaints of voice changes or dysphagia.

**FIGURE 1:**
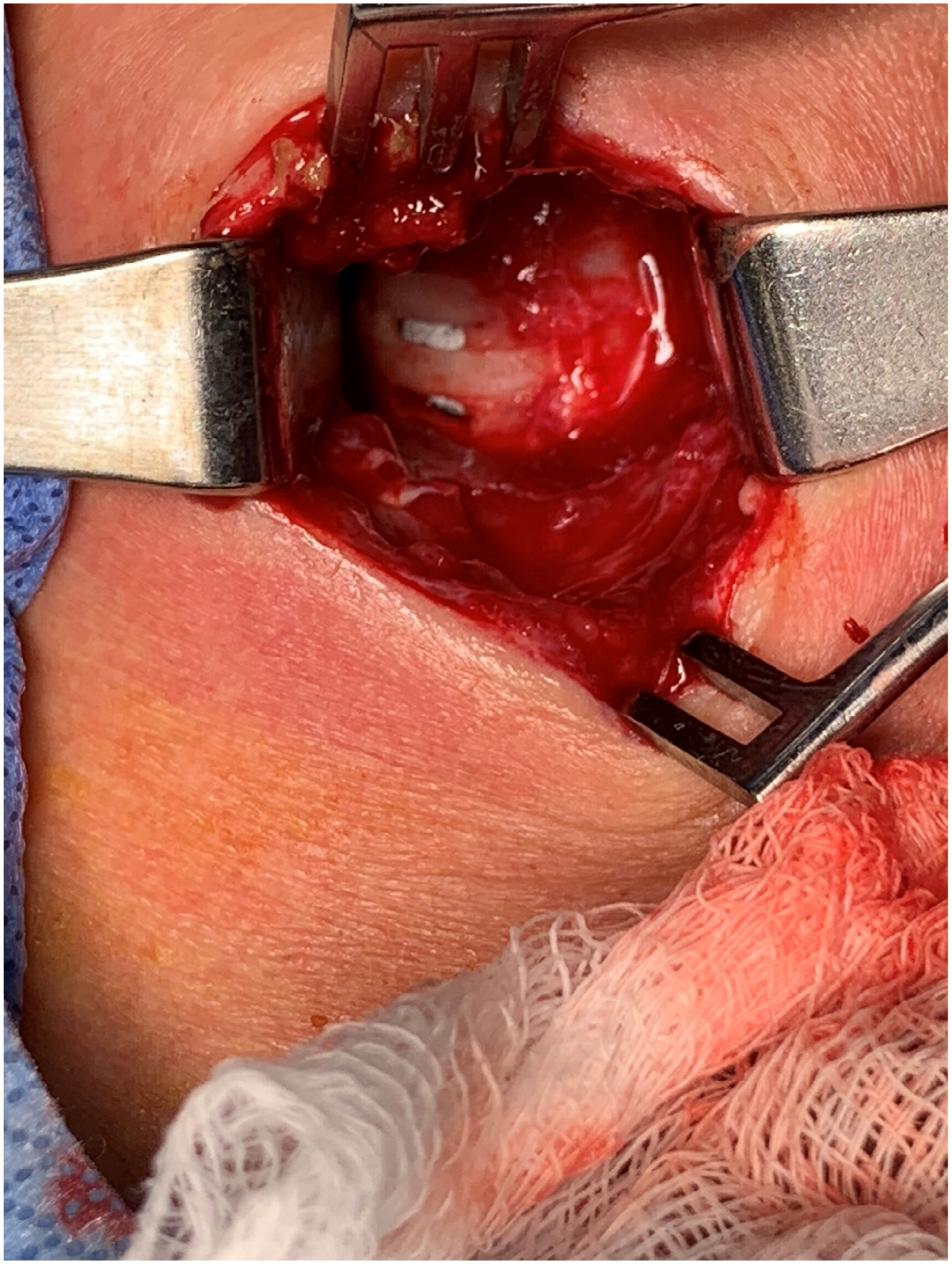
Necrosis of tracheal rings 2–4 8 days following completion thyroidectomy

The patient was taken to the operating room for wound exploration. The wound was irrigated with saline when breakdown of tissue was noted and debrided between cartilaginous rings 2, 3, and 4 with the largest amount of breakdown between 3 and 4 as shown in Figure 1. A #6 Shiley tracheostomy tube was placed between 3rd and 4th tracheal rings, with 4–0 Ethilon nylon suture placed on either side of wound. The patient was then admitted to the hospital for observation and discharged on postoperative day 3. One week following discharge, she was seen in the clinic for follow‐up; her tracheostomy tube was downsized to a #4 Shiley tracheostomy tube. Using a Passy‐Muir tracheostomy swallowing and speaking valve, the patient was able to phonate well with a mild amount of air escape in her neck.

Two weeks later, approximately 1 month since the completion thyroidectomy, she was taken to the OR for direct laryngoscopy with rigid bronchoscopy. Findings revealed mild subglottic stenosis with no signs of tracheal necrosis and was subsequently decannulated. Over the following 2–3 months, the stoma closed appropriately with no further signs of a tracheocutaneous fistula or symptoms of dysphagia, dysphonia, or stridor. She did have postoperative I‐131 radioactive ablation without issue.

## 3 DISCUSSION

The formation of a tracheocutaneous fistula secondary to tracheal necrosis following a thyroidectomy may be secondary to several etiologies. It has been previously reported following radiation therapy,^1^ iatrogenic tracheal injury, prolonged intubation following the procedure, infection of surgical site, excessive cautery on tracheal wall,^2^ or pressure‐induced ischemia.^3^ Many surgeons attempt to minimize excessive cautery around the trachea to prevent necrosis, using cautery on a lower setting when necessary.^2^ Some authors report tracheomalacia as a potential cause of tracheal necrosis as thyroid tissue may act as framework for the trachea, causing tracheal collapse when removed.^4^

Presenting symptoms of tracheal necrosis tend to occur within the first week following insult and may include shortness of breath, cough, stridor, symptoms of mediastinitis including chest pain, and formation of a tracheocutaneous fistula.^5^ Treatment for tracheal necrosis has not been standardized due to the rarity of the complication. Treatment options depend on the severity of necrosis and number of segments involved. For small defects, strap musculature may be rotated over the dehiscence for soft tissue covering, and for larger defects, a pectoralis flap may be utilized.^6^ In cases of tracheitis, wound vacuum therapy has been reported.^7^ A conservative approach was taken with this patient given patient follow‐up reliability, maintaining pectoralis major myofascial flap as a possible future intervention should wound fail to heal.

In our case, we postulate the formation of a seroma postoperatively potentially led to a pressure‐induced necrosis of the anterior tracheal wall. Only one case has been reported to our knowledge of tracheal necrosis in the setting of a postoperative seroma. The pathogenesis of a seroma‐induced pressure necrosis is likely similar to the hypothesized manner in which elevated cuff pressure leads to tracheal necrosis. The hypothesis is the cuff pressure exceeds perfusion pressure of tracheal mucosa, resulting in pressure necrosis.^8^ The other manner in which tracheal necrosis may have occurred is due to disrupting the blood supply to the tracheal wall. Blood supplying the trachea stems from the inferior thyroid artery. These branches can be very delicate and easily interrupted with cauterization during the removal of thyroid tissue.^8^ The largest risk factor in this patient for tracheal necrosis would be the need for a second procedure for the complete removal of thyroid tissue. Compared with a single procedure, a two‐step procedure would increase the amount of time intubated and lead to repeat use of cautery around anterior tracheal wall. The thyroid and trachea both receive blood supply from the inferior thyroid arteries; these were meticulously ligated at the distal branches immediately prior to entering thyroid tissue. However, some branches supplying the trachea may have been inadvertently damaged during the procedure.

Other common risk factors for tracheal necrosis that were not a factor in our patient include postoperative infection, large thyroid goiter that might have compromised tracheal blood supply, marked bleeding during the procedure leading to excessive cauterization for hemostasis, and prior chemoradiation.^5^

## 4 CONCLUSION

In retrospect, a single‐stage procedure as well as a more aggressive postoperative follow‐up course may have prevented this complication. Risks of performing a hemithyroidectomy in place of a complete thyroidectomy during initial procedure were discussed with the patient who elected to assume these risks. However, the need for a second procedure may have led to the formation of tracheal necrosis. The patient reported noticing seepage from her incision for 1–2 days prior to the follow‐up appointment wherein she had 14 cc of fluid aspirated from the seroma; had she followed up sooner, there may have been less pressure‐induced compromised blood supply to the tracheal wall.

## AUTHOR CONTRIBUTIONS

TC and CS were involved in reviewing the literature and preparing and editing the manuscript, and the authors approved the final version of the manuscript. CS was involved in the treatment of the patient.

## CONFLICT OF INTEREST

None declared.

## ETHICAL APPROVAL

This study does not require any ethical committee approval.

## CONSENT

Written consent was obtained from the patient to publish this report in accordance with the journal's patient consent policy.

## Data Availability

The data that support the findings of this study are available from the corresponding author upon reasonable request.
